# The anti-clot treatment scale (ACTS): validation of the translated Arabic version among patients undergoing warfarin therapy in Saudi Arabia

**DOI:** 10.1186/s12955-020-01471-4

**Published:** 2020-07-06

**Authors:** Sireen Abdul Rahim Shilbayeh, Alnada Abdalla Ibrahim

**Affiliations:** Department of Pharmaceutical Practice, College of Pharmacy, Princess Nourah Bint Abdulrahman University, P.O. Box 84428, Riyadh, 11671 Saudi Arabia

**Keywords:** Oral anticoagulant, Patient-reported outcomes, Arabic version, Treatment satisfaction, Warfarin, Psychometric validation

## Abstract

**Background:**

Long-term anticoagulation therapy, particularly with warfarin, is usually associated with poor adherence and low patient satisfaction. However, previous studies have highlighted the possibility that individual perceptions of warfarin differ according to cultural practices. This study validated the psychometric properties of the translated Arabic version of the Anti-Clot Treatment Scale (ACTS) for patients on warfarin therapy in Saudi Arabia.

**Methods:**

A cross-sectional multicenter study was conducted at the three main medical centers in Riyadh. Stratified sampling was employed to recruit Arabic-speaking patients who had been taking warfarin for a minimum of 3 months for any indication. The patients completed the specific ACTS along with the generic Treatment Satisfaction Questionnaire for Medication (TSQM 1.4) at two clinic visits. The psychometric performance of the ACTS was evaluated using well-established criteria: feasibility, reliability, and validity.

**Results:**

One hundred thirty-six patients participated in the study (mean age: 50.68 ± 14.6 years; range: 19–97). Overall, the patients reported moderate Burdens and Benefits scores (44 ± 9.9 and 11.92 ± 2.4, respectively) compared to the reference range for each subscale (12–60 and 3–15, respectively); however, they reported lower Burdens scores than other populations. Consistent with the original ACTS validation study, the criteria for acceptability (data targeting, floor/ceiling effects, and skewness) were satisfied; in fact, the Arabic version exhibited better item- and scale-level distributions of data than versions in other languages. The ACTS subscales also demonstrated satisfactory test-retest reliability with significant intraclass correlation coefficients ((ICC ≥ 0.5); *p* < 0.001) and good internal consistency (all Cronbach’s alpha values exceeded 0.7). Exploratory factor analysis supported the 2-factor loading model. Interestingly, the Arabic version exhibited greater convergent validity with the TSQM subdomains (r = 0.61).

**Conclusions:**

This study provides convincing evidence that the Arabic versions of both the ACTS Burdens and ACTS Benefits scales are equivalent to other versions in terms of psychometric performance, as measured using reliability and validity criteria. These properties support the great potential of the Arabic ACTS to accurately reflect patient satisfaction, identify aspects of treatment that need improvement in clinical practice, and compare treatment satisfaction across different anticoagulant therapies or cultures in research.

## Background

Long-term anticoagulation therapy, particularly with warfarin and other vitamin K antagonists (VKAs), is mostly indicated for the prevention of recurrent venous thromboembolism (VTE) complications [1]. Recurrent VTE is known to increase morbidity and mortality rates, with further negative effects on health care resources and patient quality of life [2]. Therefore, patient persistence with anticoagulation treatment for more than 3 months is crucial to maintain the therapeutic goal (an international normalized ratio, or INR, of 2–3) and mitigate the increased risk of VTE recurrence [3]. However, patients’ adherence was reported to decrease over the course of treatment due to their concerns about the risk of anticoagulant-associated bleeding complications and the burdens of frequent monitoring, dietary restrictions and dose adjustment [4, 5].

Therefore, provider concerns about patient compliance with anticoagulant regimens are likely to limit the widespread adoption of the indicated anticoagulation therapy [6].

However, a previous review of observational studies examining anticoagulation therapy has highlighted possible variability in individual perceptions of barriers to adherence and in associated lifestyle experiences according to psychosocial factors, attitudes and cultural practices [7]. In addition, studies that directly addressed quality of life reported considerable variability in patients’ quality-of-life preferences regarding warfarin therapy, and their conclusions were often controversial [8, 9].

Previous national surveys in Saudi Arabia revealed that patients’ nonadherence scores were high [10–15] but consistent with other populations internationally [16, 17]. Attempts to formally quantify the effects of anticoagulants on quality of life and patient satisfaction were have been very limited and have often used unvalidated tools [11, 12]. Researchers have recommended further studies to provide insights into adherence and reasons for discontinuation, which potentially have critical implications for improving the quality of anticoagulant care [14].

Accordingly, well-structured research within the context of anticoagulant treatment satisfaction is still required to evaluate the reasons for poor adherence and actions to increase adherence in terms of the culture of our individual patients. As a first step, an accurate estimation of pertinent components and values contributing to anticoagulant treatment satisfaction using robust, validated tools is necessary.

The Anti-Clot Treatment Scale (ACTS) is a patient-reported instrument including several of the items most relevant to the burdens and benefits associated with anticoagulation therapy. This scale was specifically designed to assess the satisfaction of patients receiving long-term anticoagulation treatment, regardless of the pharmacological subtype or clinical indication for anticoagulant treatment. The ACTS has been robustly validated in multiple languages, but not Arabic [18].

Under these premises, the main aim of this study was to validate the use of the ACTS questionnaire by patients who were being treated with oral anticoagulants in Saudi anticoagulant clinics (ACCs). The secondary objective was to determine the ability of the two subscales of the ACTS (Burdens and Benefits) to discriminate among patients known to differ in their levels of INR control parameters, thus providing a solid foundation of evidence to guide decision making and improve the management of individual patients.

## Methods

### Study design and participants

A cross-sectional multicenter study was conducted in ACCs at the three main medical centers (King Khalid Medical City, Security Forces Hospital and Prince Sultan Military Medical City) in Riyadh. Participants were recruited from September 30, 2017, to December 30, 2017. Stratified sampling was employed to recruit patients who met the following criteria: (a) aged > 18 years, (b) Arabic-speaking, and (c) receiving warfarin for a minimum of 3 months for any indication. We excluded (a) patients who were < 18 or ≥ 75 years old; (b) treatment-naïve patients (i.e., oral anticoagulation treatment (OAT) had been prescribed, but not yet received); (c) patients previously diagnosed with mental disorders; and (d) patients with visual, auditory, or oral communication deficiencies.

### Procedures

Complete demographic and clinical background information was obtained for all included patients from the hospitals’ electronic medical records using a specifically designed data collection sheet. The form collected information regarding the demographic characteristics of each patient, as well as his or her indication for anticoagulation, warfarin dosage history, comorbid conditions, concurrent medications, INR values, date of INR measurement, target INR, and incidence of bleeding. Selected patients completed the study questionnaires in the ACC waiting area in the presence of the research team during their routine appointments. The questionnaires required approximately 30–60 min to complete. For the purpose of the test-retest validity evaluation, a second ACC visit (within a period of 7 to 14 days) was scheduled at the patients’ convenience to readminister the questionnaires.

### Measures

#### The ACTS

The ACTS is a patient-reported instrument that has been robustly validated as a measure of treatment satisfaction, specifically for anticoagulants [18]. This scale comprises 17 items across two subscales: Burdens (13 questions) and Benefits (4 questions). ACTS is available in a variety of languages, but not in Arabic [18]. Therefore, the scale was translated to the Arabic language according to standard guidelines [19] and after obtaining official permission. Four Arabic professors of pharmacy at Princess Nourah University judged the face and content validity of the final Arabic version of this questionnaire. The final Arabic version of the ACTS is available upon request from authors. Subscale scores were calculated in accordance with the developers’ guidelines. In this study, the ACTS was administered at two set time points, the first at 0 days and the second within 14 days.

#### Treatment Satisfaction Questionnaire for Medication (TSQM 1.4)

The TSQM 1.4 is a generic patient-satisfaction measure developed for diverse patients and medications [20]. The TSQM includes four essential domains of satisfaction with medications: effectiveness (items 1–3), side effects (items 4–8), convenience (items 9–11), and global satisfaction (items 12–14). The scores for the domains range from 0 (extremely dissatisfied) to 100 (extremely satisfied) points. The TSQM has been widely employed in diverse clinical settings [21–23] as a multilingually validated questionnaire, and the Arabic translation has been previously validated in Saudi ACCs [15].

In this study, during the ACC test visit, the patients were initially advised to respond to both the ACTS and the TSQM. At the second encounter, a subset of patients was asked to complete the TSQM along with the ACTS questionnaire to provide a benchmark for assessing the convergent validity of the ACTS score via hypothesized correlations between the ACTS and the TSQM subscales.

The final satisfaction outcome in this study was based on patients’ responses to the anticoagulation-specific ACTS questionnaire, and a supporting analysis was provided by the generic TSQM 1.4 for validation purposes.

#### INR control

The quality of INR control was quantified by two indicators: 1) the mean percentage of time in the therapeutic range (TTR; days), assessed using the method reported by Rosendaal et al. [24], and 2) the percentage of visits during which the INR readings were in the range (INR stability), which was assessed using the methodology described by Kaatz [25] and Rose et al. [26].

### Data management

All data were coded and analyzed using the Statistical Package for Social Sciences (SPSS) (version 25.0; IBM SPSS Statistics for Windows. Armonk, NY: IBM Corp.). For the descriptive evaluation of demographic and clinical data, continuous variables were reported as the means (standard deviations, SD) after the normality of their distribution was confirmed using the Kolmogorov–Smirnov test, while categorical variables were presented as proportions.

Among the data collected, the following properties of the ACTS were selected to be examined for the purpose of psychometrically validating the scale:

The *feasibility* of the ACTS items was evaluated by calculating the rate of nonresponses (proportions of missing values) and testing for ceiling and floor effects of each item and each domain (Burdens and Benefits). In addition, data targeting was assessed by computing the distribution of both ACTS subscores as the actual range divided by the possible range multiplied by 100. A fixed criterion for this assessment has not been established, but the distribution is considered more favorable when it is closer to 100%, which indicates better targeting [27, 28].

The *internal consistency* of each subscale of the ACTS (Burdens and Benefits) was assessed by individually calculating Cronbach’s alpha for each domain and determining the interitem and item-total mean correlations. The internal consistency tests were replicated for time point 1 and time point 2. The criterion for accepting Cronbach’s alpha is a score ≥ 0.7 [29–31].

*The test-retest reliability* of the ACTS subscales was assessed by calculating the intraclass correlation coefficient (ICC) based on datasets collected at two time points (time point 1 and time point 2) that were separated by 7 to 14 days, assuming that no interventions were administered within this short period that would significantly modify the patients’ treatment satisfaction.

To investigate the *construct validity* of the ACTS, we subjected the 17 items to an exploratory factor analysis (EFA) with the overarching goal of identifying the underlying relationships among the translated scale items. The appropriateness of the sample size was evaluated using the Kaiser-Meyer-Olkin (KMO) measure (required to be greater than 0.5 for sampling adequacy) [32]. In addition, Bartlett’s test was used to identify whether the correlations between items were sufficiently large for a factor analysis to be applied. The analysis employed principal component extraction and varimax rotation with Kaiser normalization methods.

The *convergent validity* of the ACTS domains (Burdens and Benefits) was assessed by determining their correlations with scores on the TSQM and its four subdomains (effectiveness, side effects, convenience, and global satisfaction) using the Spearman rank-order correlation coefficients.

A *known-group validity analysis* was conducted to determine the ability of the two subscales of the ACTS (Burdens and Benefits) to discriminate among patients known to differ in their levels of INR control parameters. We anticipated that individuals who were more satisfied with their medication were likely to exhibit a better anticoagulation control status. The association of ACTS subscale (Burdens and Benefits) scores with INR control subclasses was first examined using the Wilcoxon signed-rank test and then the binary logistic regression analysis. The model specified INR control (controlled, uncontrolled) as the outcome and ACTS as the score. Since the ACTS includes two domains, each was used separately as a determinant of INR control in a simple univariate binary logistic regression model. Subsequently, the ACTS domains that were significantly related to INR control (*p* < 0.05) were examined together in a multivariate binary logistic regression model that included INR control after controlling for patient covariates (such as age, gender, and education level) that were significantly related to treatment satisfaction in the criterion validity analysis.

## Results

### Descriptive analysis of the study sample

One hundred thirty-six patients met the inclusion criteria and participated in the study. The mean age was 50.68 years (SD, 14.6). Overall, the participants were primarily female (*N* = 97; 71.3%). The majority of participants (58%) had either attained a high school (30.1%) or diploma/university education (27.2%), while only 6 (14.7%) had not received any formal education. At the time of recruitment, the patients had been taking warfarin for an average of 3.6 years (SD = 3.2), and their average TTR score was at the poor control level (< 60%; mean = 58.8; SD = 33.6). However, the patients’ mean INR stability value was within the “safe warfarin management” status (≥ 50%; mean = 63.5%; SD = 35). The most common warfarin indication was atrial fibrillation (*N* = 31; 22.8%), followed by prosthetic heart valve replacement (*N* = 29; 21.3%).

### Observed ACTS and TSQM scores

Overall, the mean (SD) score for the ACTS subscale as of the first visit (test) was in the acceptable range (compared to the reference range provided by original ACTS authors) for both Burdens and Benefits (44 ± 9.9 and 11.92 ± 2.4, respectively). As of the second visit (retest), a good level of satisfaction was also reported; however, a minimal change was observed in both domains (44.8 ± 9.7 and 12 ± 2.3, respectively) compared to the prior visit.

Regarding the TSQM tool employed in the first visit, the mean (SD) domain scores were 72.9 (14.4) for effectiveness, 86.8 (23.9) for side effects, 69.2 (15.3) for convenience, and 70.3 (14.4) for global satisfaction. At the retest visit, TSQM data were collected from 44 patients and displayed no significant changes in any of the four domains compared to the first visit.

### ACTS feasibility

As shown in Table 1, no missing data were identified at either the item level or the scale level. This completeness reflects the high data quality. Accordingly, mean scores for scales were generated for 100% of the participants. The distribution of the scores was excellent: 100% for each individual ACTS item, 94% for the Burdens scale and 92% for the Benefits scale. The investigation of floor/ceiling effects (%) showed lower values for the Burdens scale (0/2) than for the Benefits scale (0/21). However, the skewness statistic for all data was within the acceptable limits (− 1 to + 1).

**Table 1:** Feasibility of the ACTS Burdens and Benefits scales, *N* = 136

	Items with missing data (%)	Possible range (midpoint)	Actual score range	Distribution (%^**a**^)	Mean score ± SD	Floor/ceiling effects (%/%)^**b**^	Skewness
**Burden items**							
Item 1	0	1–5 (3)	1–5	100	3.51 ± 1.29	10/29	−0.454
Item 2	0	1–5 (3)	1–5	100	3.70 ± 1.30	7/39	−0.582
Item 3	0	1–5 (3)	1–5	100	3.62 ± 1.30	8/34	−0.550
Item 4	0	1–5 (3)	1–5	100	3.71 ± 1.37	9/42	−0.636
Item 5	0	1–5 (3)	1–5	100	3.52 ± 1.25	9/27	−0.467
Item 6	0	1–5 (3)	1–5	100	3.71 ± 1.19	7/33	−0.643
Item 7	0	1–5 (3)	1–5	100	3.46 ± 1.21	9/24	−0.421
Item 8	0	1–5 (3)	1–5	100	3.52 ± 1.41	12/38	−0.425
Item 9	0	1–5 (3)	1–5	100	3.88 ± 1.30	7/47	−0.862
Item 10	0	1–5 (3)	1–5	100	3.64 ± 1.28	8/34	− 0.585
Item 11	0	1–5 (3)	1–5	100	3.93 ± 1.18	4/45	−0.807
Item 12	0	1–5 (3)	1–5	100	3.82 ± 1.25	6/43	−0.707
**ACTS Burdens scale**	0	12–60 (36)	15–60	94	44.01 ± 9.89	0/2	−0.556
**Benefits items**							
Item 14	0	1–5 (3)	1–5	100	4.14 ± 0.93	1/43	−0.903
Item 15	0	1–5 (3)	1–5	100	3.91 ± 0.94	2/31	−0.586
Item 16	0	1–5 (3)	1–5	100	3.87 ± 1.04	4/33	−0.737
**ACTS Benefit scale**	0	3–15 (9)	4–15	92	11.91 ± 2.39	0/21	−0.556

^a^Calculated as the actual range divided by possible range multiplied by 100

^b^Calculated as the percentage of people scoring either 12 (floor) or 60 (ceiling) on the ACTS Burdens scale or 3 (floor) or 15 (ceiling) on the ACTS Benefits scale

*Abbreviaton*: *SD* standard deviation

### Reliability of the ACTS

#### Internal consistency and reliability

As described in Table 2, on the item level, all the item-total correlations were greater than 0.4 (range: 0.37–0.75) for all 15 items at time point 1 and time point 2, indicating a high level of internal consistency.

**Table 2:** Internal consistency of the ACTS domains and items

Domains and items	Time point 1, ***N*** = 136		Time point 2, ***N*** = 129	
	Item-total correlation	Cronbach’s alpha	Item-total correlation	Cronbach’s alpha
**Burden scale**				
Item 1	0.375	0.874	0.453	0.9
Item 2	0.468	0.868	0.517	0.897
Item 3	0.366	0.874	0.518	0.897
Item 4	0.566	0.862	0.701	0.888
Item 5	0.491	0.866	0.563	0.895
Item 6	0.634	0.858	0.602	0.893
Item 7	0.715	0.853	0.564	0.895
Item 8	0.572	0.862	0.653	0.89
Item 9	0.599	0.86	0.735	0.886
Item 10	0.648	0.857	0.694	0.888
Item 11	0.667	0.856	0.753	0.885
Item 12	0.662	0.856	0.703	0.887
ICC (95% CI of the ICC)			0.786 (0.671–0.836), *p* < 0.001	
**Benefit scale**				
Item 14	0.523	0.587	0.391	0.771
Item 15	0.523	0.587	0.604	0.5
Item 16	0.485	0.635	0.577	0.541
ICC (95% CI of the ICC)			0.500 (0.293–0.646), *p* < 0.001	

*Abbreviation*: *ICC* intraclass correlation coefficient

Additionally, on the scale level, the two subscales appeared to display good internal consistency, with all Cronbach’s alpha values exceeding 0.7. The total Cronbach’s alpha values at time point 1 and time point 2 were 0.87 and 0.9 for the Burdens scale and 0.695 and 0.702 for the Benefits scale, respectively.

#### Test-retest reproducibility

One hundred twenty-nine patients were available and completed the ACTS questionnaire again during the second visit (average 11.2 ± 2 days). All 15 items exhibited moderate correlations (0.3–0.7) at the two time points (Table 1). The test-retest ICCs for both the ACTS Burdens and ACTS Benefits scores were acceptable (ICC ≥ 0.5), revealing a significant correlation between the test and retest scores (*p* < 0.001 in both sets). However, the ICC of the Burdens scale (0.79) was higher than the Benefits scale (0.5).

### Validity of the ACTS

#### Construct validity

**Fig. 1:**
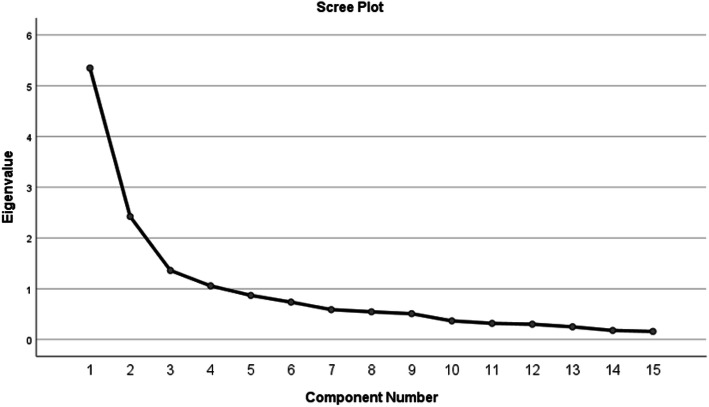
Scree plot of components of the ACTS Arabic version

The adequacy of the sample size was evidenced by a KMO value of 0.828. In addition, Bartlett’s test showed a significant result (χ^2^ = 1046.84, *p* < 0.001), suggesting that correlations within the R-matrix were significantly different from zero and hence justified the use of the factor analysis. First, four factors were extracted based on Kaiser’s criterion of retaining factors with eigenvalues greater than 1 (the last reported eigenvalue was 1.24). A scree plot (Fig. 1) showed a clear break after two data points. This break represented a two-factor solution that explained 51.80% of the total variance (30.10% for the Burdens scale and 21.70% for the Benefits scale). The values of item loadings in the rotated component matrix were all greater than 0.5 (Table 3).

**Table 3:** Construct validity of the ACTS Burdens and Benefits scales – Arabic version:, *N* = 136

Item	Components	
	Component 1	Component 2
Item 1	0.584	−0.050
Item 2	0.564	.0001
Item 3	0.554	−0.138
Item 4	0.749	−0.159
Item 5	0.644	−0.020
Item 6	0.624	−0.366
Item 7	0.613	−0.194
Item 8	0.754	0.012
Item 9	0.815	−0.048
Item 10	0.760	−0.095
Item 11	0.820	−0.107
Item 12	0.784	−0.092
Item 14	0.089	0.692
Item 15	−0.258	0.813
Item 16	−0.128	0.820

Extraction method: principal components analysis; rotation method: varimax with Kaiser normalization

Values represent factor loadings on each component: Component 1 = ACTS Burdens subscale; Component 2 = ACTS Benefits subscale

#### Convergent validity

Table 4 displays the results of the convergent validity analysis, as expressed by the Spearman’s rho coefficients between the ACTS subscales and the scores of the total TSQM questionnaire and its four subdomains. As expected, all correlations between the ACTS Burdens scale and TSQM subdomains, except for the domain of side effects, were positive, moderate (0.3–0.7), and statistically significant (*p* < 0.01). Similar correlations were observed between the ACTS Benefits score and all TSQM subdomains. These correlations indicated an association between high scores on the specific ACTS questionnaire (greater satisfaction with warfarin) and a high degree of general treatment satisfaction measured using the TSQM questionnaire. Interestingly, the strongest correlation was observed between the ACTS Burdens subscale and the TSQM convenience domain (r = 0.61) and between the ACTS Benefits subscale and the TSQM effectiveness satisfaction score (r = 0.58).

**Table 4:** Convergent validity of the ACTS Burdens and Benefits subscales – correlations with TSQM (total and subscales), *N* = 42

Domains	ACTS Burdens	ACTS Benefits	TSQEFF	TSQSIDE	TSQCON	TSQGLO	TSQM Total
**ACTS Burdens**	1.000						
**ACTS Benefits**	0.402**	1.000					
**TSQEFF**	0.473**	0.581**	1.000				
**TSQSIDE**	0.240	0.380*	0.201	1.000			
**TSQCON**	0.605**	0.328*	0.530**	0.215	1.000		
**TSQGLO**	0.420**	0.557**	0.619**	0.330*	0.578**	1.000	
**TSQM Total**	0.620**	0.486**	0.794**	0.320*	0.848**	0.843**	1.000

*Abbreviations*: *ACTS* Anti-Clot Treatment Scale, *TSQEFF* TSQM Effectiveness, *TSQSIDE* TSQM Side effects, *TSQCON* TSQM Convenience, *TSQGLO* TSQM Global Satisfaction

*The correlation is significant at *p* < 0.05

**The correlation is significant at *p* < 0.01

#### Known-groups validity

Table 5 depicts the comparisons of clinically discernable known groups of INR control parameters in terms of their responses to both subscales of the ACTS (Burdens and Benefits). Surprisingly, the analysis did not reveal statistically significant associations, contradicting our expectation of the a priori hypothesis that individuals who are more satisfied with their anticoagulant therapy would be more adherent and consequently more likely to exhibit a good coagulation control status.

**Table 5:** Known-group validity of ACTS subscales by INR control categories

Subscale	Moderate to optimum TTR (≥60%)	Poor TTR (< 60%)	AOR (95% CI)	***p***-value
**Burdens**	45.4 ± 8.8	42.8 ± 10.6	0.97 (0.92–1.02)	0.19
**Benefits**	12.4 ± 2.6	11.5 ± 2.3	0.85 (0.7–1.03)	0.08
**Subscale**	**Optimum INR stability (**≥ 50%)	**Poor INR stability (**< 50%)	**AOR (95% CI)**	***p*-value**
**Burdens**	44.3 ± 10.4	44.4 ± 9.7	0.96 (0.9–1.02)	0.98
**Benefits**	11.8 ± 2.5	11.8 ± 2.4	0.99 (0.8–1.24)	0.98

Note: *TTR* time in the therapeutic range, *AOR* adjusted odds ratio per 10-unit increase

## Discussion

This study was conducted with the primary aim of validating and reporting the psychometric properties of a treatment-specific satisfaction assessment tool, the ACTS, in patients undergoing warfarin therapy. Unlike a previous international study of the original ACTS, which involved patients with a diagnosis of acute symptomatic VTE [18], this study analyzed patients with various long-term indications for anticoagulant therapy. In addition, this study addressed the practicality of using the translated Arabic version of the ACTS in future studies that aim to evaluate satisfaction with other new oral anticoagulant therapies that have recently started to be prescribed in the Saudi population. The ACTS was selected because it has been validated in different languages for different cultural groups [18, 33] and has been employed as an outcome in large-scale, gold-standard randomized controlled trials (RCTs) involving patients with atrial fibrillation [33, 34] or VTE [35, 36].

This study provided good evidence for the feasibility of using the ACTS, with good data quality in the form of 100% patient completion rates for the ACTS Burdens and ACTS Benefits subscales. Similar to the original ACTS validation study [18], other indicators of acceptability, including data targeting (as assessed by the score distributions), floor/ceiling effects, and skewness statistics, were all acceptable. In fact, the ACTS Arabic version used in the current study showed better targeting, particularly regarding the absence of skewness in the item- and scale-level data, than the versions in other languages, which displayed slight positive skewness in the score distributions of all items of the Burdens subscale (except items 7 and 10; all countries; sk ranged from − 1.22 to − 2.19) and the total Burdens subscale (English and French versions; sk = − 1.12 and sk = − 1.35, respectively) and total Benefits subscale (German version, sk = − 1.97) [18].

Additionally, the translated Arabic version of the ACTS exhibited acceptable internal consistency; however, the value of Cronbach’s alpha was higher for the ACTS Burdens subscale (0.9) than for the ACTS Benefits subscale (0.7). These high alpha values indicate the reliability of the scale for general application among patients receiving warfarin therapy for various indications in the ACC-managed Saudi population. This outcome is comparable to other versions of ACTS (Cronbach’s alpha values ranged from 0.79 to 0.9) [18, 33, 35]. However, other versions (Dutch, Italian, French, German, English, and Spanish language versions) were specifically validated in patients who had venous thromboembolism [18] or patients with atrial fibrillation [33] and who were treated with either warfarin or other newer oral anticoagulants. Other forms of reliability, such as scale-level test-retest reproducibility, were also confirmed in the current study, with a significant ICC between total scores for the Burdens and Benefits subscales at both clinic visits, confirming that the ACTS is a stable tool for evaluating treatment satisfaction in patients who have not been exposed to any new clinical intervention or modification of anticoagulant therapy between the test and retest responses. This result supported the consistency of patient-reported Benefits and Burdens scores over time observed with other ACTS versions, regardless of the anticoagulant therapy received [18, 33, 35]. In particular, this psychometric property indirectly adds to the already great potential for using the ACTS tool in long-term RCTs to compare patient satisfaction with different therapies [35, 36].

Regarding the validity, the results of the construct validity analyses conducted in this study were consistent with those of the original version [18] in confirming the grouping of the ACTS items into two subscales (Burdens and Benefits) and validating their correlations with the corresponding subdomains. Based on these results, items in each subscale measured a shared principal construct and contained a comparable amount of information. Interestingly, the findings of the convergent validity analysis revealed a significant positive correlation (r-values ranging from 0.33 to 0.61, *p* < 0.01) between the ACTS subscales and total scores, as well as with most subdomain scores of the general treatment satisfaction tool (TSQM Arabic version), which was previously validated in the patient population of Saudi ACCs [15]. These results differed from the data obtained in the original validation study [18], where the degrees of association between both ACTS subscale scores and the TSQM domains were less than expected (r = 0.27–0.35). However, the nonsignificant correlation between the ACTS Burdens subscale and the TSQM side effects domain in the current evaluation was consistent with the original validation study [18]. A potential explanation for these results is that TSQM side effects items do not explicitly examine the critical anticoagulation-specific side effects of bleeding and bruising, which are appraised in ACTS Burdens items [18]. Another study reported significant positive correlations between the ACTS questionnaire (Spanish version) [33] and the Self-Assessment of Treatment Question (SAT-Q) and EuroQol (EQ-5D) questionnaires, both of which are intended to assess the level of general, but not specific, patient satisfaction with treatment [37].

Regarding known-group validity, this study failed to identify any significant correlation between ACTS satisfaction scores and the gold-standard INR control parameters. This finding is partially explained by two concurrent facts: the average poor INR control (TTR < 60%; mean = 58.8) observed in the current population and their long duration of warfarin use (3.6 years SD = 3.2), reflecting higher than expected satisfaction scores due to adaptation to their therapy. This correlation with the clinical examination was not addressed in the original validation study [18]. In contrast, our findings in the previous validation study of the Arabic TSQM quite interestingly revealed that moderate to optimal INR control was significantly and independently associated with increased satisfaction scores for the domains of effectiveness and convenience, but not side effects or global satisfaction [15]. Therefore, the explicit ability of the ACTS Burdens and Benefits subscales to detect clinically meaningful changes within patients and differences across various anticoagulant therapies remains to be elucidated in long-term follow-up research studies.

To the best of our knowledge, this study is the first to report a full psychometric analysis in Arabic-speaking patients for an instrument specifically measuring satisfaction with anticoagulant treatment. The study confirmed the robustness of the ACTS in measuring satisfaction, despite cultural or environmental discrepancies in patient groups. However, our analysis has some limitations.

The first limitation of this study is its cross-sectional design, particularly due to its use of a self-reported measure, which might be confounded by temporary circumstances that bias measures and serve as sources of common method variance. Any of these confounders might limit the generalizability and consistency of findings obtained through this design compared to longitudinal studies [38]. However, the distribution of the scores was excellent at both the item and scale levels. In addition, the test-retest reproducibility in the current study confirmed the stability of ACTS scores over time. Additionally, in contrast to the original validation study that only included patients with a specific disease [18], the variability of patients’ fixed variables (demographics, concurrent illnesses, and anticoagulant indications) might improve the generalizability of our results and limit the potential of bias arising from the cross-sectional design [38].

Second, this study analyzed a small sample compared to previous international studies, although the sample size was satisfactory for the purposes of psychometric validation. Nonetheless, the small number of patients might limit the predictive power of factors associated with satisfaction among individual patients (i.e., insufficient numbers of patients might have been included in the subgroup analysis). Further prospective multicenter studies should be conducted to confirm the results obtained in the present study and obtain a better understanding of the variables that improve satisfaction with anticoagulant therapy.

A final limitation of our study is that head-to-head comparisons between patients’ preferences for warfarin and nonwarfarin therapy were not performed due to limited availability and use of other alternative treatments in this cohort. Therefore, future studies should focus on exploring the potential to modify satisfaction scores by comparing patients’ opinions based on their experience with newer alternative anticoagulant agents.

## Conclusions

In conclusion, this study provides convincing evidence supporting the psychometric validity of the Arabic version of the ACTS as a feasible measure of satisfaction with anticoagulant therapy in patients visiting ACCs. In fact, the psychometric performance of the Arabic versions of both the ACTS Burdens and ACTS Benefits subscales is equivalent to versions published in other languages in terms of fulfilling traditional psychometric criteria for data quality, targeting, reliability and validity. Therefore, this tool is expected to produce a validated patient-reported outcome that accurately reflects patients’ satisfaction with anticoagulant therapy, which will be helpful in clinical practice for identifying aspects of treatments needing improvement and in research studies comparing satisfaction with different therapies. Thus, favorable aspects of patients’ experiences with their medications would be captured on a common scale across different cultures. However, a further evaluation of satisfaction in a prospective longitudinal study is warranted to confirm the robustness of the ACTS Burdens and Benefits subscales in detecting subsequent alterations in the gold-standard clinical parameters.

## Data Availability

The datasets used and/or analyzed during the current study are available from the corresponding author upon reasonable request.

## Abbreviations

ACC: Anticoagulant clinic
ACTS: Anti-Clot Treatment Scale
ICC: Intraclass correlation coefficient
INR: International normalized ratio
KMO: Kaiser-Meyer-Olkin
M: Mean
OAT: Oral anticoagulation treatment
PCA: Principal component analysis
SD: Standard deviation
TSQM: Treatment Satisfaction Questionnaire for Medication
TTR: Time in the therapeutic range
VKA: Vitamin K antagonist
VTE: Venous thromboembolism
